# Inter-agency Coordination for Rural Power Restoration After A Natural Disaster in the United States: A Qualitative Interview Dataset

**DOI:** 10.1038/s41597-026-06994-x

**Published:** 2026-03-17

**Authors:** Rui Shao, Kalyan Piratla, Chien-fei Chen, Mark Abuanor, Chao Fan

**Affiliations:** 1 College of Engineering, Computing, and Applied Sciences, Clemson University, Clemson, SC 29631 USA; 2 Department of Sociology, Anthropology and Criminal Justice, Clemson, SC 29631 USA

## Abstract

In September 2024, Hurricane Helene inflicted unprecedented damage across South Carolina’s Upstate and nearby inland counties in North Carolina, causing widespread, prolonged power outages that disproportionately affected rural communities and demanded tightly coordinated action among public utilities, government agencies, and nongovernmental organizations to restore electric service. To capture time-sensitive lessons from this response, we collected a qualitative dataset on interagency coordination during power-restoration operations. This data descriptor presents a set of in-depth, semi-structured interviews conducted in 2025 with key personnel directly involved in the Upstate South Carolina restoration including 21 managers from utilities, governmental bodies, and community organizations. The interviews probe organizational adaptation, interagency alignment, and the emergence of team flow in coordinated restoration. The dataset is intended to help researchers and policymakers understand the complex, ad hoc collaboration that underpins disaster recovery and to provide qualitative context that complements quantitative analyses of infrastructure resilience.

## Background & Summary

In September 2024, Hurricane Helene struck Upstate South Carolina, an inland and largely rural region historically unprepared for direct hurricane impacts, causing unprecedented damage and economic losses^1^. More than 90% of residents lost power, and restoration in some areas took up to two weeks, driven largely by extensive treefall on saturated soils^2^. The event exposed significant resilience vulnerabilities, especially in heavily wooded, resource-constrained rural communities. Ad hoc collaboration among resource-limited rural agencies during disaster response remains poorly understood in the literature. Helene and the subsequent restoration provided a critical context to collect empirical evidence on critical-infrastructure recovery, particularly electric power, covering restoration timelines and the factors shaping multi-organization coordination^3^.

This paper presents a dataset of in-depth, semi-structured interviews with key personnel involved in post-Helene restoration efforts^4,5^. The dataset captures perishable information on organizational behavior, decision-making, and teamwork to highlight challenges in rural settings and inform more effective interagency operations during critical-infrastructure restoration. The interviews explored:

- Challenges and successes of inter-agency communication and coordination^6^
- Processes of organizational adaptation and ad-hoc restructuring during crisis response^7–9^;
- Factors influencing the emergence of team flow and its impact on restoration efficiency^10^; and,
- Rural-specific barriers to disaster recovery, including resource constraints and infrastructure vulnerabilities^11^.

The dataset stems from a large-scale research project*, Data Collection on Electric Power Restoration Timelines and Factors Affecting Them in the Aftermath of Hurricane Helene in Upstate South Carolina Region*, funded by the National Science Foundation (CMMI-2503593) in 2024. The program proceeds in two phases: (1) in-depth qualitative interviews to capture perishable data on team hurdles, organizational restructuring, and collaboration protocols; and (2) data assimilation and preliminary analysis to identify key themes and barriers to effective collaboration and recovery in rural communities.

Subsequent work will advance quantitative survey and modelling methods that integrate these findings to reflect rural organizational and sociotechnical characteristics, enabling understanding of temporal recovery under varying conditions, such as treefall, transportation constraints and household response. The dataset reported here is the principal output of Phase 1 and documents the experiences and perspectives of practitioners who managed and executed recovery across Upstate South Carolina.

This dataset provides a sociotechnical foundation for deploying emerging resilience technologies like mobile renewable microgrids. It moves beyond technical specifications to reveal the real-world operations that determine the success or failure of new infrastructure in a crisis. By documenting these human and organizational dynamics, the interviews offer an empirical basis for the interdisciplinary co-design of effective deployment strategies and policies, ensuring future resilient clean energy solutions are both technologically advanced and operationally viable^12^.

## Methods

The complexity of the disaster situation and the difference in how specific organizational and individual behaviors are restructured under emergency conditions cannot be captured through large-scale survey instruments alone, particularly community-level data. Responses to, and outcomes from, a major crisis are likely to differ significantly based on organizational context, available resources, and pre-existing relationships. Hence, a qualitative method such as interviews was developed to collect perishable data on organizational adaptation, multi-agency alignment, and team flow in the coordinated restoration of electric power supply^4^. The qualitative interviews focus on issues of theoretical and policy interest, particularly the concept of team flow, which is defined as the intense experiential involvement in moment-to-moment activity that optimizes collaboration and maximizes a team’s performance^10,13^. A major objective of the interviews was to deeply understand how the prerequisites for team flow, such as a collective ambition, a common goal, high-skill integration, and open communication, emerge within ad-hoc teams of crews with different skills and from different regions, especially in the context of emergency response in rural communities. It was also intended that the understandings gleaned from the interviews would inform the development of future models for predicting power restoration timelines.

### Data collection procedures

Interviews were conducted remotely via videoconference between June and August 2025 by researchers from Clemson University (CU). CU’s Institutional Review Board approved the study in March 2025 prior to data collection under the record number IRB2025-0290. All methods of this study were performed in accordance with the relevant guidelines and regulations. Participants were informed that interviews would be recorded for anonymized transcription and analysis, and all 21 interviewees provided consent.

This 9–11 months post-event timeframe was selected to capture a specific type of perishable data, given the operational realities of power restoration. The informal, ad hoc coordination strategies that are frequently omitted from formalized After-Action Reports are lost as organizations solidify their official narratives. Furthermore, as the reconstruction of major utility infrastructure often extends beyond a year, this window allowed participants to provide reflective accounts of the immediate crisis while still actively engaged in the long-term recovery process, ensuring that operational details remained fresh and less prone to recall bias.

### Participants and Sampling

This paper targeted four groups of leaders, managers, and practitioners who played critical roles in the Hurricane Helene recovery. Participants were recruited via purposive, word-of-mouth outreach initiated by trusted community stakeholders to capture diverse organizational perspectives on the restoration process^5,12^. Sample sizes and selection criteria varied by groups:

1. Utilities (n = 8): Managers from electric cooperatives, privately owned utilities, and municipal water/sewer authorities, selected to provide frontline perspectives on infrastructure damage, restoration logistics, and resource allocation.
2. Government agencies (n = 8): State and county emergency management, public works, and local government leaders, selected to speak to macro-level coordination, Emergency Operations Center (EOC) activities, and policy execution.
3. Regulatory/coordination officials (n = 2): State-level officials overseeing utility regulation and the broader energy-restoration response, selected to provide a statewide view of priorities and resource mobilization.
4. Community and nonprofit organizations (n = 3): Leaders engaged in volunteer coordination and community support, selected to capture grassroots perspectives on mutual aid and gaps in the official response.

Our sample distribution reflects the different operational structures of organizations involved. In practice, functionally flatter organizations like community groups often consolidate multiple roles into a single leader, whereas more hierarchical entities like utility companies feature a greater division of labor, necessitating a larger sample to capture their full perspective^14–16^. The variation in sample sizes is designed to reflect the distinct roles and organizational structures within the recovery process rather than to achieve numerical parity across groups.

### Interview questions development

Drawing on prior disaster literature, theories of social order, crisis communication, multi-level resilience, and the multi-level nature of post-disaster response where stakeholders hold distinct yet interdependent roles, we employed semi-structured interviews to capture perishable, fine-grained information that large-scale surveys cannot easily observe, particularly at the community level^17–20^.

In practice, after reviewing initial post-disaster reports and previous disaster literature, the research team prioritized questions highlighted by Helene’s specific challenges and by recurrent themes in the literature. The final protocol probed how participants’ organizational roles, communication protocols, and collaborative experiences shaped response efforts. Interviews focused on four core themes; Table 1 lists each theme alongside the corresponding key lines of questioning.

**Table 1:** Interview topics and line of questioning.

Theme	Line of Questioning	Targeted Team Flow Prerequisite
**Roles & Responsibilities**	• Organization’s mission & individual’s role • Influence on power restoration • Most significant disaster impacts	**Autonomy & skill integration** (Understanding one’s specific contribution to the collective system)
**Communication & Coordination**	• Organizational restructuring • Challenges with out-of-state crews • situational awareness issues • Public communication & misinformation	**Immediate feedback & open communication** (The availability of real-time performance feedback)
**Logistics & Resource Allocation**	• Immediate actions after disaster • Allocation of critical resources (fuel, equipment, generator) • Role of Mutual Aid Agreements	**Shared goals** (Alignment of operational targets across agencies)
**Teamwork & Lessons Learned**	• Inter-agency learning & innovation • Success factors & bottlenecks in collaboration • Specific recommendations for the future	**Trust & familiarity** (Prerequisites for rapid synchronization)

Instead of asking participants directly about subjective flow states, which are concepts that can be abstract and difficult for non-psychology practitioners to articulate. The protocol employed a strategy of contextualized questioning to capture rich, situational evidence. The design focuses on probing the presence or absence of the theoretical prerequisites for team flow as defined by Jana and Richard (2018), such as shared goals and immediate feedback^21^. By grounding inquiries in specific operational scenarios rather than abstract constructs, we elicited detailed narratives regarding the structural conditions of the response. As outlined in Table 1, this approach ensures that the dataset captures the nuanced details necessary to determine whether the conditions for flow were met, providing a context-rich, flexible evidence for diverse analytical interpretations.

### Interview procedure

All interviews were conducted remotely via video conference in June–August 2025. We followed a team-based, semi-structured format: each session was led by the Principal Investigator, with one or more co-investigators and graduate research assistants present, enabling multidisciplinary observation and note-taking^4,5,22^. Procedures were standardized. Sessions began with introductions and a brief statement of project objectives, followed by verbal informed consent. Participants received a digital information sheet in advance outlining the study scope, potential risks and benefits, data-retention policies, and IRB approval details. The consent terms specified that:

- Participation was voluntary and could be withdrawn at any time;
- Any question could be declined;
- Sessions will be audio-recorded for transcription purposes only, and recordings will be destroyed upon project completion;
- All published data will be anonymized to protect confidentiality.

Upon recording verbal consent, the interview proceeded using a semi-structured protocol organized around four themes (see Table 1). The guide balanced consistency across interviews with flexibility for follow-up and probing, allowing the team to capture nuanced, emergent features of ad hoc disaster response and to maximize the value of perishable qualitative evidence. Most interviews lasted 45–90 minutes, with variation reflecting participant communication styles and iterative refinement of questions as thematic saturation was approached within each target group.

### Composition of the final dataset

This dataset comprises 21 interviews with leaders, managers, and practitioners from organizations involved in Hurricane Helene recovery across Upstate South Carolina. Table S1 summarizes interviewee roles by organization type; Table 2 reports the distribution of participants across key functional areas of the restoration effort. The descriptions of participant roles in Table Table S1 have been standardized into a *Tier (Core Function)* format. Specifically, the *Senior Management* tier encompasses roles such as executives, directors, and chiefs; the *Managerial* tier includes managers and administrators; and the *Operation* tier covers supervisors and field-level staff. This adjustment was made to protect the anonymity of the interviewees, as the specific names of roles risk being identifiable.

**Table 2:** Quantitative distribution of participants across key recovery functions.

Primary Function	Number of Participants Involved
Strategic Command & EOC Operation	10
On-Site Infrastructure Restoration	9
Logistics & Resource Coordination	10
Public & Inter-Agency Communication	12
Community & Volunteer Support	5

Designed to capture a multi-sector, multi-level view of electric-power restoration, the 21 participants represent 19 distinct roles across six organization types: electric utilities, water utilities, municipal government, state agencies, universities, and nonprofit/community organizations. This structure brings together perspectives from strategic decision-makers to frontline operations and logistics. Two roles are represented by two interviewees each, yielding a total of 21 participants.

Functionally, the most frequently represented responsibilities were Public & Inter-Agency Communication (n = 12), Logistics & Resource Coordination (n = 10), and Strategic Command & EOC Operations (n = 10). Accordingly, the dataset is particularly rich in material on coordination, communication, and command—providing field evidence relevant to “team flow” dynamics in rural disaster management. The cumulative count across functions (46) exceeds the number of interviewees (21) because many participants held responsibilities spanning multiple functional areas, reflecting the overlapping, cross-team nature of disaster response and power-restoration work.

## Data Records

The Hurricane Helene Recovery Interview Dataset is deposited on Harvard Dataverse^23^. The dataset comprises three key components: (1) interview transcripts: a collection of verbatim records of the 21 de-identified interview transcripts. (2) the interview protocol contains a PDF document outlining the guide used during data collection, including core questions and thematic prompts that guided the conversation, and (3) The metadata file consists of an XLSX spreadsheet containing anonymized spreadsheet summarizing participant roles, their corresponding organization types, and aggregate data on the sampling distribution across the four target groups. Table 3 provides an overview of the files available through the selected repository^23^.

**Table 3:** File names and types as stored on the data repository.

File Name	File Type	Notes
**01_Helene_Interview_Transcripts.zip**	.zip	A compressed folder containing all 21 de-identified interview transcripts.
**02_Helene_Interview_Protocol.pdf**	.pdf	The interview protocol and question guide.
**03_Helene_Metadata.xlsx**	.xlsx	Supporting metadata file with participant and organization details.

## Technical Validation

To ensure the dataset’s quality, reliability, and ethical integrity, we implemented a multi-step validation process. First, all interview audio was transcribed by a professional third-party service and then double-checked by the research team against the recordings to correct errors and standardize terminology^24^. Second, transcripts were manually de-identified to remove direct and potentially identifying details (e.g., personal names, agency names, precise locations), consistent with the informed-consent agreement and IRB approval. Lastly, an initial round of thematic analysis using the qualitative data analysis software MAXQDA confirmed the corpus’s richness and relevance to the project’s core research questions. The coding process employed a hybrid framework, combining deductive codes derived from theory with inductive themes emergent from the data, with at least two team members reviewing coding consistency and resolving discrepancies through a negotiated consensus approach^16,25,26^. We verified that the semi-structured protocol was applied consistently across all 21 interviews and elicited detailed responses across the intended themes. Furthermore, the sample size was validated by monitoring for thematic saturation, which was confirmed when later interviews demonstrated informational redundancy. When reinforcing established patterns without yielding new high-level themes, it supports the comparability and the internal coherence of the dataset.

## Usage Notes

### Practical research application

Beyond simple recovery records, this qualitative dataset enables a detailed examination of the sociotechnical dynamics of disaster restoration. An illustrative use case conducted on 10 sample transcripts investigates how the disruption of team flow prerequisites, specifically obstacles to effective information exchange, from immediate feedback loops to collective ambition, acts as a primary bottleneck in rural power restoration (See Appendix A). By applying a coding approach that identifies the absence or failure of these conditions, researchers can quantify the impact of physical infrastructure damage on psychological and organizational synergy. For instance, preliminary analysis reveals that in 90% of the cases, the physical destruction of communication hardware in mountainous terrain cuts off power. It subsequently severed the immediate feedback loops, which are essential for team flow, thereby stalling inter-agency coordination. Furthermore, the results also point out the importance of aligning different teams’ priorities. This analysis demonstrates how the dataset enables research teams to integrate qualitative interview evidence with quantitative measures, providing a basis for testing theories of organizational behavior. It can also provide practical evidence for investment in infrastructure resilience in the concrete context of rural recovery operations.

### Data access process

This dataset is publicly available on Harvard Dataverse^23^. Users can access and download the interview transcripts, protocol, and metadata directly without administrative restrictions. To protect participant confidentiality, all data have been strictly de-identified in accordance with IRB requirements prior to publication.

Users can create an account by visiting https://dataverse.harvard.edu/, selecting the Sign-up option, and following the on-screen instructions. After registration, they should access the dataset directly via 10.7910/DVN/XJ4CPZ^23^.

### Limitations

While the snowball sampling strategy successfully identified key decision-makers, it inherently introduces selection bias, as referrals tend to remain within existing professional networks. Therefore, the dataset predominantly reflects the perspectives of utility operators and emergency managers. The voices of end-users and lower-level contractors are less represented, which may limit the dataset’s applicability for researching community-level psychological impacts or purely technical component-level failure analysis.

As noted in the Data Collection section, interviews were conducted 9–11 months post-event to capture reflective institutional memory. We acknowledge that this temporal gap inevitably introduces a degree of recall bias regarding precise timestamps or minute-by-minute sequencing of events. While we verified key dates against public records, the granular details of ad hoc conversations are based on participants’ retrospective reconstructions and should be interpreted accordingly.

The data collection was conducted by academic researchers with backgrounds in sociology, engineering and disaster management. On one hand, it may have encouraged participants to explain technical concepts more explicitly than they would to an industry peer, thereby enhancing data richness for a broader audience. On the other hand, participants may have potentially understated internal conflicts to present to their organizations in a favorable way. To mitigate this, the interviewers employed neutral probing techniques and emphasized the study’s focus on systemic learning rather than totally absorbing individual statements.

## Supplementary information

Appendix

Supplementary Information Table

## Data Availability

The inter-agency coordination dataset, including interview transcripts, protocol, and metadata, is publicly available on the Harvard Dataverse repository (10.7910/DVN/XJ4CPZ).

## Code availability

No custom code has been used for the interview dataset presented in this paper.
