# Supplementary material for: Inter-agency Coordination for Rural Power Restoration After A Natural Disaster in the United States: A Qualitative Interview Dataset
Source: Sci Data. 2026 Mar 17;13:692. doi: 10.1038/s41597-026-06994-x (PMC13139420; doi:10.1038/s41597-026-06994-x)
Supplement: Supplementary file 1 — Appendix [file 41597_2026_6994_MOESM1_ESM.docx]

**Appendix A: Analyzing barriers to team information flow in rural disaster response**

This analysis uses ten interview records from the dataset to investigate how the failure of information coordination mechanisms, such as quick feedback and shared goals, slows down power restoration. The study focuses on how these mechanisms, which usually keep agencies working together, are interrupted by the socio-technical effects of a hurricane.

The study examined 10 randomly selected transcripts to identify specific moments when cooperation failed, as shown in Table A1. The records were examined to identify instances in which participants mentioned elements that prevented agencies from obtaining the information they needed to make decisions or hindered their ability to agree on which tasks to do first.

**Table A1. Mapping information flow failures**

| Theoretical Prerequisite | Theme | Codes | Frequency (n=10) | Illustrative Quote |
| --- | --- | --- | --- | --- |
| Immediate feedback | Feedback loop breakdown | Physical comms failure | 9/10 | “We were operating in a total vacuum... but by and large we weren't sure if they were energized or not until someone drove back.” |
|  |  | Information lag | 8/10 | “The biggest challenge was the time delay between the field assessments and the data reaching the EOC due to signal shadows.” |
| Collective ambition | Goal misalignment | Priority conflict | 9/10 | “The town wanted the streets opened, but the utility needed to wait for de-energizing lines; this misalignment created a standstill.” |
|  |  | Resource competition | 5/10 | “Different counties had different agendas for which substation was critical, leading to friction in mutual aid allocation.” |
| Open comms | Information silos | Informal path dependency | 3/10 | “We didn’t have a shared dashboard; I had to call the utility manager’s personal cell just to know where their crews were.” |
| Skill integration | Integration friction | Local knowledge gap | 2/10 | “Outside crews had the technical skill but lacked the terrain familiarity, causing a gap in integrating their work into our flow.” |

Through thematic analysis and quantification, this study identified several clear obstacles to coordination through information sharing. One primary obstacle is the physical break in the feedback loop between the field and the office. In an interview with a utility manager, the participant noted that because there was no cell signal, the dispatch center was in an information vacuum for the first part of the response. They had to wait for workers to drive back to the office to give a verbal report before they knew which substations were damaged. This physical need to travel to share information led to delays in decision-making. This situation was common in interviews in mountainous areas, where hills blocked radio and phone signals, delaying field notes from reaching managers.

Another obstacle is the difference in priorities between organizations. In one case, city workers wanted to clear debris from the roads immediately, but the power company needed to wait until they were certain the lines were not energized before allowing anyone near the debris. When these two goals occurred in the same place, and there was no shared communication platform to resolve the order of operations, work stalled. The interview records show that this lack of a shared plan for which task comes first used up a lot of time during the first few days of the recovery.

In addition, the research shows that when formal ways of sharing information failed, workers had to rely on personal relationships to get things done. In one interview, a local leader said that because there was no shared computer dashboard showing where crews were, he had to call a manager at the power company on their personal cell phone. This reliance on knowing someone personally shows that when technical tools break down, social connections are the only way to keep the restoration moving.

These findings have practical meaning for disaster planning. They show that in rural areas, simply having more repair crews is not enough if the physical environment still prevents information from moving between them. If the feedback loop is broken, the efficiency of the whole group drops. This analysis of the interviews demonstrates the value of the dataset by showing how researchers can use a mixed-methods approach to connect socio-technical environmental factors to organizational behavior. It can serve as a basis for developing better disaster restoration plans and for more realistic modeling of coordination behavior in rural communities.
